# Acupuncture and herbal medicine for postoperative care following anterior cruciate ligament reconstruction: A protocol for systematic review and meta-analysis

**DOI:** 10.1097/MD.0000000000024936

**Published:** 2021-02-26

**Authors:** Hyungsuk Kim, Won-Seok Chung

**Affiliations:** Department of Clinical Korean Medicine Graduate School, Kyung Hee University, 23 Kyungheedae-ro, Dongdaemun-gu, Seoul, Korea.

**Keywords:** acupuncture, anterior cruciate ligament reconstruction, herbal medicine, protocol, systematic review

## Abstract

**Background:**

Anterior cruciate ligament reconstruction (ACLR) is the primary treatment for patients with anterior cruciate ligament (ACL) injury. Successful postoperative rehabilitation is imperative for their recovery. This protocol details the methods that will be used to systematically analyze the efficacy of acupuncture and herbal medicine for postoperative care following ACLR.

**Methods and analysis:**

Randomized controlled trials will be searched in the following databases: the Cochrane Central Register of Controlled Trials (CENTRAL), EMBASE, MEDLINE/PubMed, Chinese National Knowledge Infrastructure, Japan Medical Abstracts Society, and 7 Korean databases (Oriental Medicine Advanced Searching Integrated System, Korean National Assembly Digital Library, Korean Association of Medical Journal Editors, Korean Studies Information Service System, Korean Traditional Knowledge Portal, National Digital Science Library, and Database Periodical Information Academic). The risk of bias will be assessed using the Cochrane assessment tool of risk of bias. The studies that are selected after checking for eligibility will be quantitatively analyzed as a meta-analysis. The primary outcome will be the scores of pain scales, and the secondary outcomes will be the range of motion of the knee, severity of the swelling, and parameters about the knee joint function.

**Ethics and dissemination:**

Ethical approval is not required for this protocol because it does not include patient data. The findings of this review will be disseminated through peer-reviewed publications or conference presentations.

**Registration number:**

DOI 10.17605/OSF.IO/ZY2W8 (https://osf.io/zy2w8).

## 1 Introduction

The anterior cruciate ligament (ACL) plays an important role in stabilizing the knee joint during movements and is the most easily injured structure among victims of knee trauma. It is related to movements such as pivoting, jumping, and changing direction during sports activity. Approximately 250,000 patients suffer from an ACL injury, of which 120,000 individuals undergo surgery for anterior cruciate ligament reconstruction (ACLR) every year in the United States.^[1]^ Rehabilitation following ACLR is the key for the patient to return to normal activities. It includes rebuilding the muscles and restoring proprioception around the knee area.^[2,3]^ Although there is some consensus about the best rehabilitative approach after ACLR, there is still controversy about the best rehabilitative approach for the patient to return to the previous activities and sports.^[4]^

Traditional Chinese medicine (TCM), which comprises conservative approaches such as acupuncture and herbal medicine, has been shown to be effective for rehabilitation in various postoperative conditions, including total knee arthroplasty^[5]^ and spine surgery.^[6]^ However, there are no systematic reviews regarding the efficacy of acupuncture and herbal medicine in the rehabilitative stage after ACLR, although the use of these complementary therapies is gaining popularity among practitioners, and many randomized controlled trials (RCTs) have been reported.^[7,8]^ This review, for the first time, will systematically analyze the efficacy of acupuncture and herbal medicine in patients who underwent ACLR.

## 2 Methods

### 2.1 Study registration

This systematic review protocol follows the preferred reporting items for systematic reviews and meta-analysis (PRISMA) protocols.^[9]^ The protocol of this study has been registered in the Open Science Framework (osf.io/zy2w8).

### 2.2 Eligible criteria for study selection

#### 2.2.1 Types of studies

Since the aim of this study is the outcomes of synthesis of the data derived by a parallel study design, only RCTs will be included. Crossover trials and quasi-RCTs will not be included. There will be no limitations to language. Ongoing research, as well as published studies, will be searched.

#### 2.2.2 Types of participants

Patients who were diagnosed with an ACL injury and underwent ACLR will be included. Patients who have undergone other surgeries or those with severe comorbidities or complications after ACLR will be excluded.

#### 2.2.3 Types of interventions

Intervention in the form of acupuncture or herbal medicine will be considered in this review. The combination of acupuncture or herbal medicine with other TCM treatment methods, such as moxibustion, fumigation, massaging along the meridian, and fuming-washing therapy, will be considered as the intervention group. TCM combined with rehabilitation will also be included if identical treatment methods were used for both the intervention and control groups. Any type of acupuncture involving electric or heat stimuli that penetrates the skin will be included. Acupuncture without skin penetration, such as laser acupuncture and acupressure, will not be considered in this study. All orally administered herbal medicines will be included regardless of the number of kinds or types of herbs. The comparison group will comprise patients who underwent conventional rehabilitation such as physiotherapy, analgesics, and patient education. If only both experimental and comparison groups have the same control treatment, the study will be included for this review. A study with acupuncture or herbal medicine therapy as a control group will not be included since the purpose of this review is to compare acupuncture or herbal medicine with other treatments.

#### 2.2.4 Types of outcome measures

The primary outcome will be the scores of pain scales, including the visual analog scale and numerical rating scale. The secondary outcomes will be the range of motion of the knee, the severity of the swelling, and a parameter that documents the function of the knee joint, including parameters such as the Lysholm score, International Knee Documentation Committee 2000 subjective knee form, and Hospital for Special Surgery score.

### 2.3 Search strategy

#### 2.3.1 Electronic data

The following databases will be searched from their inception until June 2020: The Cochrane Central Register of Controlled Trials (CENTRAL), EMBASE, MEDLINE/PubMed, Chinese National Knowledge Infrastructure, Japan Medical Abstracts Society, and 7 Korean databases (Oriental Medicine Advanced Searching Integrated System, Korean National Assembly Digital Library, Korean Association of Medical Journal Editors, Korean Studies Information Service System, Korean Traditional Knowledge Portal, National Digital Science Library, and Database Periodical Information Academic). The search strategy for PubMed is presented in Table 1.

**Table 1:** Search strategies for PubMed.

#1	anterior cruciate ligament reconstruction [MeSH]
#2	anterior cruciate ligament injuries [MeSH]
#3	“anterior cruciate ligament repair” [tw]
#4	“anterior cruciate ligament” [tw]
#5	#1 OR #2 OR #3 OR #4
#6	“anterior cruciate ligament” [tw]
#7	“intra-articular knee ligament” [tw]
#8	#6 OR #7
#9	injury OR rupture OR torn OR destruction OR trauma OR reconstruction OR repair
#10	#8 AND #9
#11	#5 OR #10
#12	acupuncture therapy [MeSH]
#13	Acupuncture [MeSH]
#14	acupuncture point [MeSH]
#15	“acupuncture needle” [tw]
#16	Meridians [MeSH]
#17	acupuncture^∗^[tw]
#18	needle^∗^[tw]
#19	acupoint^∗^[tw]
#20	Electroacupuncture [MeSH]
#21	electro-acupuncture [tw]
#22	pharmacoacupunctur^∗^[tw]
#23	pharmaco-acupunctur^∗^[tw]
#24	“acupoint injection”[tw]
#25	“auricular acupunctur^∗^”[tw]
#26	“ear acupunctur^∗^”[tw]
#27	“auricular needl^∗^”[tw]
#28	“ear needl^∗^”[tw]
#29	“scalp acupuncture^∗^”[tw]
#30	“fire acupunctur^∗^”[tw]
#31	“warm acupunctur^∗^”[tw]
#32	“warm needl^∗^”[tw]
#33	#12 OR #13 OR #14 OR #15 OR #16 OR #17 OR #18 OR #19 OR #20 OR #21 OR #22 OR #23 OR #24 OR #25 OR #26 OR #27 OR #28 OR #29 OR #30 OR #31 OR #32
#34	Medicine, Chinese Traditional [MeSH]
#35	Chinese herbal medicine[tw]
#36	Chinese medicine[tw]
#37	Chinese herbal drug[tw]
#38	traditional herbal medicine[tw]
#39	herbal medicine[tw]
#40	decoction[tw]
#41	tang[tw]
#42	^∗^tang[tw]
#43	formula[tw]
#44	#34 OR #35 OR #36 OR #37 OR #38 OR #39 OR #40 OR #41 OR #42 OR #43
#45	#33 OR #44
#46	“randomized controlled trial”[Publication Type]
#47	“randomized controlled trials as topic”[MeSH]
#48	“random allocation”[MeSH]
#49	“double-blind method”[MeSH]
#50	“single-blind method”[MeSH]
#51	Placebo [MeSH]
#52	random^∗^[tw]
#53	rct[tw]
#54	rct's[tw]
#55	rcts[tw]
#56	placebo^∗^[tw]
#57	#46 OR #47 OR #48 OR #49 OR #50 OR #51 OR #52 OR #53 OR #54 OR #55 OR #56
#58	#11 AND #45 AND #57

#### 2.3.2 Search for other resources

To find other literature from various sources, the references of the selected articles will be screened by titles and abstracts. Literature that is not published online will be manually searched.

### 2.4 Data collection and analysis

#### 2.4.1 Study selection

**Figure 1:**
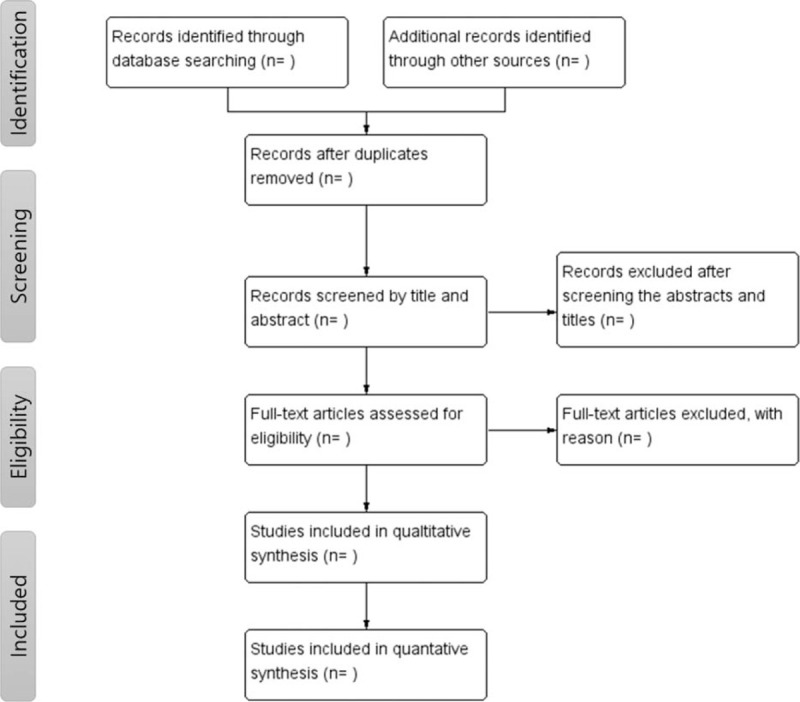
Flowchart of the review.

Two reviewers will independently search for articles according to the search strategy and the inclusion and exclusion criteria. In case of disagreement between the reviewers, other authors will discuss and make a decision. A flowchart of the selection process according to the PRISMA flow chart is presented in Figure 1.

#### 2.4.2 Data extraction and management

Data will be extracted by 2 independent reviewers using an extraction form after reading the full text. This form will include characteristics of participants, the onset of ACL injury, the date of ACLR surgery, types, times, frequency of intervention, comparator group, type and evaluation period of outcome measurements, and adverse events. In case of any agreement, another reviewer will make a decision.

#### 2.4.3 Assessment of the risk of bias and quality of included studies

The risk of bias for included studies will be evaluated by 2 independent reviewers using the tool for assessing the risk of bias in randomized trials from the Cochrane Collaboration tool.^[10]^ The domains covers 7 areas of assessments: random sequence generation, allocation concealment, blinding of participants and personnel, blinding of outcome assessment, incomplete outcome data, selective outcome reporting, and other sources of bias. Three levels of assessments will be used: high risk, low risk, or unclear risk. If there is no consensus between the 2 reviewers, other reviewers will decide the level of risk.

#### 2.4.4 Measurement of the treatment effect

For the analysis of continuous data, the mean differences (MDs) with 95% confidence intervals (CIs) will be used. When the same scale is used, weighted mean differences will be used, whereas if not the same indicators are used, standardized mean differences will be used.

#### 2.4.5 Management of missing data

When inadequate or missing data are detected, the corresponding authors will be contacted through e-mail for more information. If adequate information is not accessible, the data will not be considered in the analysis.

#### 2.4.6 Data synthesis

A meta-analysis will be conducted to synthesize the data of included studies according to measurement outcomes using the Cochrane Collaboration software (Review Manager Software Version 5.3). A fixed-effects model or a random-effects model will be used according to the level of heterogeneity. If high heterogeneity is detected (*I*^2^ > 50%), a random-effects model with 95% CI will be used. Else, a fixed-effects model will be used. Cochrane Higgins *I*^2^ greater than 75% indicates considerable heterogeneity, and subgroup analysis will be additionally conducted to interpret the outcomes and compare groups divided by the reviewers’ decision.

#### 2.4.7 Subgroup analysis

A subgroup analysis will be performed according to the following criteria, to explain the considerable heterogeneity:

1. type of treatments (i.e., acupuncture alone, herbal medicine alone, acupuncture plus herbal medicine, acupuncture plus more than 1 other TCM treatment, herbal medicine plus more than 1 other TCM treatment, acupuncture plus herbal medicine with more than 1 other TCM treatment);
2. treatment period (< 2 weeks, 2–4 weeks, 4–7 weeks, 7–8 weeks, more than 12 weeks).

The classification for the duration of treatments will be established by referring to the rehabilitation stage after ACLR.^[11]^

#### 2.4.8 Ethics and dissemination

Ethical approval is not required for this article because it does not include the private clinical information of patients. The results of this systematic review will be disseminated to journals or used for presentation at a conference.

## 3 Discussion

ACLR following an ACL injury is common, and successful rehabilitation is necessary for the resumption of normal activities. Acupuncture and herbal medicine as options for conservative care after ACLR is popularly used among clinicians for better outcomes. The efficacy of these modalities for pain relief and improvement in function has been studied previously.^[12–15]^ However, no systematic review has been conducted to determine the effect and safety of these treatments in patients undergoing ACLR. This review will help practitioners understand the efficacy of acupuncture and herbal medicine for these patients and apply these methods for optimal rehabilitation.

## Author contributions

**Conceptualization:** Won-Seok Chung.

**Data curation:** Hyungsuk Kim, Won-Seok Chung.

**Formal analysis:** Hyungsuk Kim.

**Funding acquisition:** Won-Seok Chung.

**Project administration:** Hyungsuk Kim, Won-Seok Chung.

**Writing – original draft:** Hyungsuk Kim.

**Writing – review & editing:** Hyungsuk Kim, Won-Seok Chung.
